# Crystal structure and Hirshfeld surface analysis of (aqua-κ*O*)(methanol-κ*O*)[*N*-(2-oxido­benzyl­idene)threoninato-κ^3^ *O*,*N*,*O*′]copper(II)

**DOI:** 10.1107/S2056989020011706

**Published:** 2020-08-28

**Authors:** Natsuki Katsuumi, Yuika Onami, Sayantan Pradhan, Tomoyuki Haraguchi, Takashiro Akitsu

**Affiliations:** a Department of Chemistry, Faculty of Science, Tokyo University of Science, 1-3 Kagurazaka, Shinjuku-ku, Tokyo 162-8601, Japan; b Chemical Sciences Division, Saha Institute of Nuclear Physics, 1/AF, Bidhannagar, Kolkata 700-064, India

**Keywords:** Schiff base complex, copper, amino acid, Hirshfeld analysis, crystal structure

## Abstract

In the title complex mol­ecule, [Cu(C_11_H_11_NO_4_)(CH_4_O)(H_2_O)], the Cu atom is coordinated in a distorted square-pyramidal geometry by a tridentate ligand synthesized from l-threonine and salicyl­aldehyde, one methanol mol­ecule and one water mol­ecule. In the crystal, the mol­ecules show intra- and inter­molecular O—H⋯O hydrogen bonds. The Hirshfeld surface analysis indicates that the most important contributions to the packing are H⋯H (49.4%) and H⋯O/O⋯H (31.3%) contacts.

## Abstract 2

The crystal structure of the amino acid Schiff base copper(II) complex with a tridentate ligand synthesized from salicyl­aldehyde, l-threonine and copper(II) acetate is reported.

## Chemical context   

Amino acid Schiff bases, which can be easily synthesized by condensation of primary amines with carbonyl components, are organic ligands having an azomethine (>C=N–) group. They play an important and diverse role in coordination chemistry (Qiu *et al.*, 2008 ▸; Li *et al.*, 2010 ▸; Xue *et al.*, 2009 ▸). On the other hand, copper has various oxidation states, of which the +2 oxidation state is the most stable. Copper ions readily form complexes and produce abundant coordination chemistry, while Schiff base–copper(II) complexes are known to increase the catalytic efficiency of redox reactions (Cozzi, 2004 ▸; Roy & Manassero, 2010 ▸).
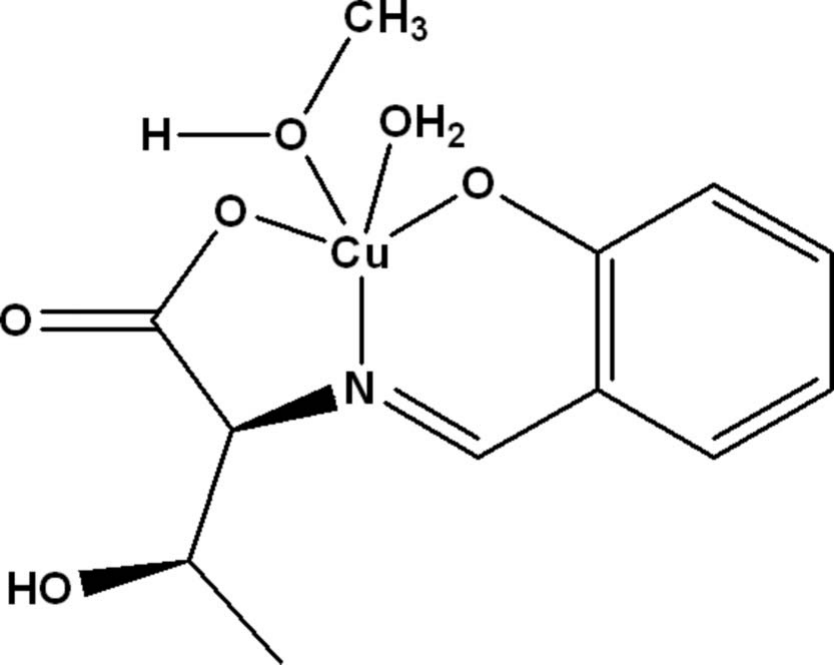


One method of reducing highly toxic Cr^VI^ compounds to less toxic Cr^III^ compounds is the use of titanium(IV) oxide, a heterogeneous photocatalyst. Although useful for such redox reactions (Kitano *et al.*, 2007 ▸; Sun *et al.*, 2006 ▸; Tuprakay & Liengcharernsit, 2005 ▸), it is only active under UV illumination (Schneider *et al.*, 2014 ▸). In our laboratory, a heterogeneous titanium (IV) oxide photocatalyst was combined with a Schiff base–Cu^II^ complex and irradiated with visible light. The presence of a π-conjugated ligand system increases the efficiency (Yoshida *et al.*, 2017 ▸; Nakagame *et al.*, 2019 ▸). It can be said that the Schiff base–copper complex has a photocatalytic effect. In the present study, the title Schiff base–copper complex was synthesized by microwave irradiation in order to shorten the synthesis time and to obtain high purity. The crystal structure is reported here.

## Structural commentary   

**Figure 1:**
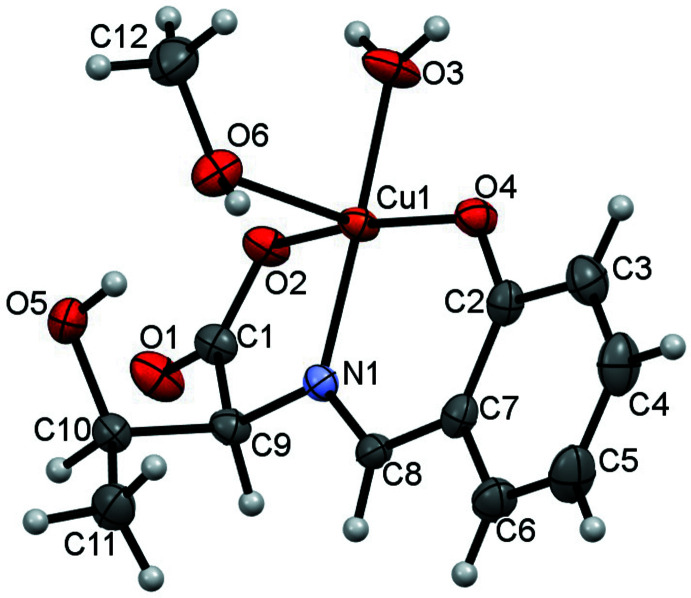
The mol­ecular structure of the title compound, showing the atom-labelling scheme. Displacement ellipsoids are drawn at the 30% probability level.

**Figure 2:**
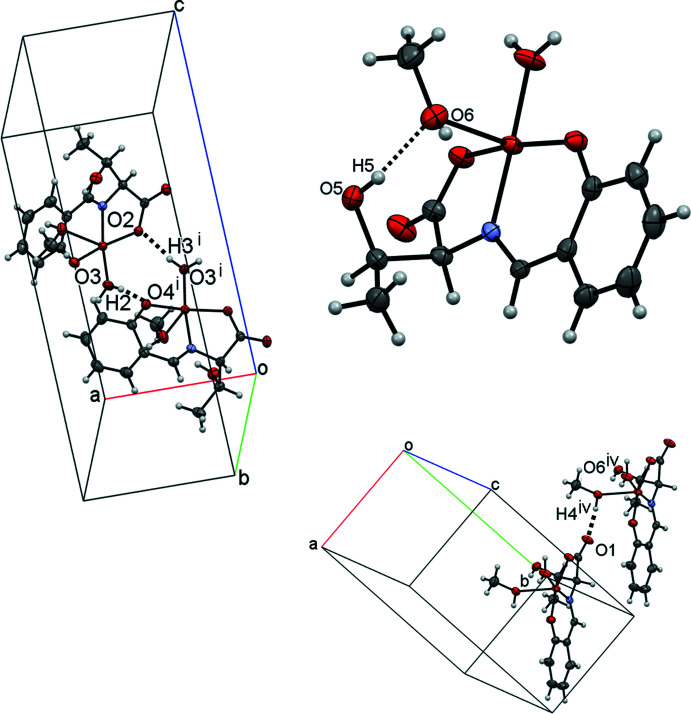
A view of the intra- and inter­molecular O—H⋯O hydrogen bonds, shown as dashed lines. [Symmetry codes: (i) *x* − 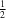, −*y* + 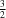, −*z* + 1; (iv) *x* − 1, *y*, *z*.]

The mol­ecular structure of the title compound consists of a tridentate ligand synthesized from l-threonine and salicyl­aldehyde, one methanol mol­ecule, and one water mol­ecule coordinating to copper (Fig. 1 ▸) in a distorted square-pyramidal coordination geometry. The C8=N1 double-bond distance is 1.286 (5) Å, close to a typical C=N double-bond length for an imine. The Cu1—O2, Cu1—O3 and Cu1—O4 bond lengths are 1.968 (3), 1.937 (3) and 1.910 (3) Å, respectively, which are close to a typical Cu—O single bond length. The Cu1—N1 bond length of 1.922 (3) Å corresponds to the typical Cu—N single-bond length. These four atoms coordinated to Cu1 have similar bond-distance values, and the contribution degree of the electron cloud is almost the same. The Cu1—O6 bond [2.471 (3) Å] has been lengthened by a pseudo Jahn–Teller effect. One intra­molecular O—H⋯O hydrogen bond (O5—H5⋯O6; Table 1 ▸) is observed between the meth­oxy function and the amino acid side chain (Fig. 2 ▸).

## Supra­molecular features   

Three inter­molecular O—H⋯O hydrogen bonds (Table 1 ▸ and Fig. 2 ▸) are observed in the crystal; one hydrogen bond (O6—H4⋯O1^iii^; symmetry code given in Table 1 ▸) forms a chain along the *a*-axis direction and while the other two hydrogen bonds (O3—H2⋯O4^i^ and O3—H3⋯O2^ii^; Table 1 ▸) form a hydrogen-bonded O2/Cu1/O3/H2/O4^i^/Cu1^i^/O3^i^/H3^i^ ring with an 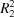
(8) motif (Table 1 ▸ and Fig. 2 ▸). The mol­ecules are stacked in a double-column along the *a*-axis direction *via* these three hydrogen bonds.

**Figure 3:**
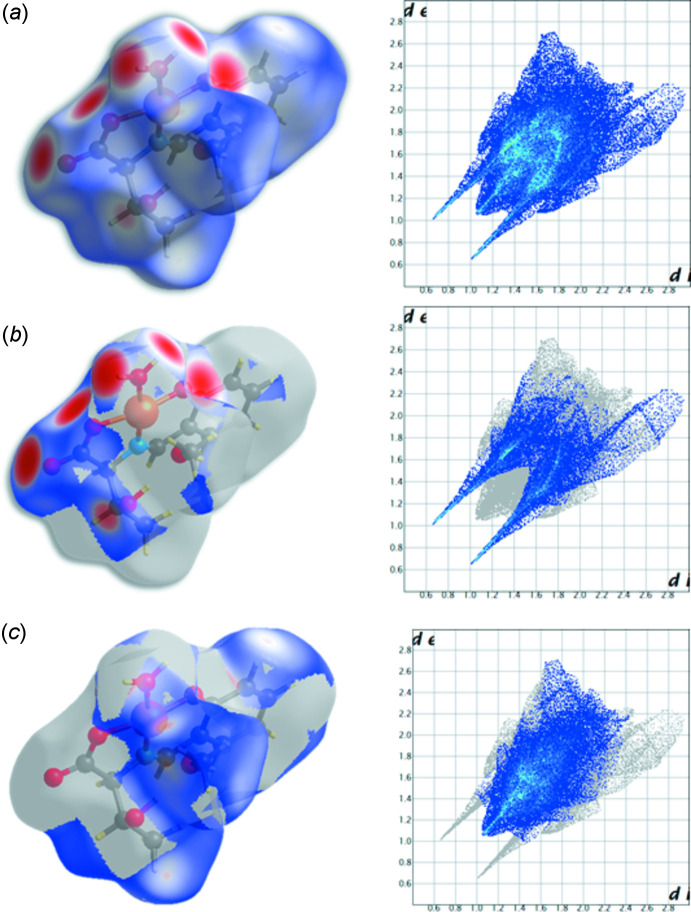
Hirshfeld surfaces mapped over *d*_norm_ (left) and two-dimensional fingerprint plots (right), showing (*a*) all inter­actions, and delineated into (*b*) H⋯O/O⋯H and (*c*) H⋯H contacts. *d*_e_ and *d*_i_ represent the distances from a point on the Hirshfeld surface to the nearest atoms outside (external) and inside (inter­nal) the surface, respectively.

**Figure 4:**
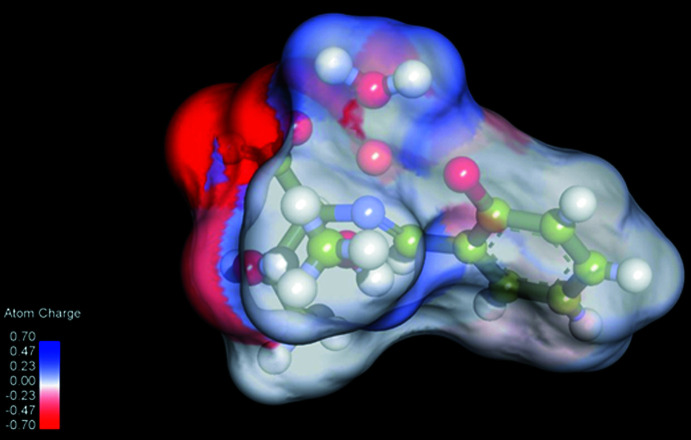
Distribution of atomic charges (red: negative, blue: positive) on the Hirshfeld surface.

Hirshfeld surface analysis (Spackman & Jayatilaka, 2009 ▸; McKinnon *et al.*, 2007 ▸) was performed to better understand the inter­molecular inter­actions and contacts. The O—H⋯O hydrogen bonds are indicated by bright-red spots appearing near O1, O2, O4 and water H atoms on the Hirshfeld surfaces mapped over *d*_norm_ and by two sharp spikes of almost the same length in the region 1.6 Å < (*d*_e_ + *d*_i_) < 2.0 Å in the 2D finger plots (Fig. 3 ▸). The contributions to the packing from H⋯H and H⋯O/O⋯H contacts are 49.4 and 31.3%, respectively. The calculated atomic charge on the surface is shown in Fig. 4 ▸. There are negative charge distributions around the O atoms of hydrogen-bond acceptors; this and other features of the inter­molecular inter­actions are in agreement with the electronegativity of atoms in the crystal structure.

## Database survey   

A search in the Cambridge Structural Database (CSD, Version 5.41, update of November 2019; Groom *et al.*, 2016 ▸) for similar structures returned three relevant entries: (2,2′-bi­pyridine-*N*,*N*′)[*N*-(2-oxido-1-naphthyl­idene)threoninato-*N*,*O*,*O*′]copper(II) (refcode BIZGIB; Qiu *et al.*, 2008 ▸), di­aqua­(*N*-salicyl­idene-l-threoninato)copper(II) (SLCDCU; Korhonen & Hämäläinen, 1981 ▸) and {*N*-[2-(hy­droxy)-3-meth­oxy­benzyl­idene]threo­nin­ato}(1,10-phenanthroline)copper hemihydrate (UQUYUB; Jing *et al.*, 2011 ▸). In the crystal of BIZGIB, a two-dimensional network is formed by a combination of inter­molecular O—H⋯O and C—H⋯O hydrogen bonds. In the crystal of SLCDCU, two mol­ecules form square planes by two inter­molecular hydrogen bonds. In the crystal of UQUYUB, inter­molecular O—H⋯O hydrogen bonds form a one-dimensional left-handed helical structure running along [001].

## Synthesis and crystallization   

l-Threonine (0.0234 g, 0.196 mmol) and salicyl­aldehyde (0.0295 g, 0.242 mmol) were dissolved in methanol (15 ml), which was treated for 5 min with microwave irradiation at 358 K to yield a transparent yellow ligand solution. To this solution, copper(II) acetate dihydrate (0.0421 g, 0.211 mmol) was added and treated for 5 min while being irradiated with microwaves at 358 K. The solution was placed in the air, and the solvent was removed. The title compound (0.0533 g, 0.169 mmol, yield 85.9%) was obtained as a green solid. IR (KBr, cm^−1^): 1633 (C=N double bond). A part of the obtained solid was dissolved in a small amount of methanol and left in air, and single crystals suitable for X-ray diffraction were obtained after several days.

## Refinement   

Crystal data, data collection and structure refinement details are summarized in Table 2 ▸. All C-bound H atoms were placed on geometrically calculated positions (C—H = 0.93–0.98 Å) and were constrained using a riding model with *U*_iso_(H) = 1.2*U*_eq_(C) for *R*_2_CH and *R*_3_CH H atoms and 1.5*U*_eq_(C) for the methyl H atoms. The O-bound H atoms were located based on a difference-Fourier map. Atoms H4 and H5 of the terminal OH group were constrained using a riding model with O—H = 0.82 Å. H5 was assigned *U*_iso_(H) = 1.2*U*_eq_(O), while the *U*_iso_ of H4 (attached to O6 was refined. Atoms H2 and H3 of the water mol­ecule were refined freely.

## Supplementary Material

Crystal structure: contains datablock(s) General, I. DOI: 10.1107/S2056989020011706/is5551sup1.cif

Structure factors: contains datablock(s) I. DOI: 10.1107/S2056989020011706/is5551Isup3.hkl

CCDC reference: 2025511

Additional supporting information:  crystallographic information; 3D view; checkCIF report

## Figures and Tables

**Table 1:** Hydrogen-bond geometry (Å, °)

*D*—H⋯*A*	*D*—H	H⋯*A*	*D*⋯*A*	*D*—H⋯*A*
O3—H2⋯O4^i^	0.87 (7)	1.84 (7)	2.692 (4)	169 (6)
O3—H3⋯O2^ii^	0.81 (6)	1.89 (6)	2.687 (4)	167 (6)
O5—H5⋯O6	0.82	1.97	2.783 (4)	171
O6—H4⋯O1^iii^	0.82	1.84	2.653 (4)	175

Symmetry codes: (i) 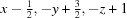; (ii) 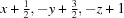; (iii) 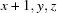.

**Table 2:** Experimental details

Crystal data	
Chemical formula	[Cu(C_11_H_11_NO_4_)(CH_4_O)(H_2_O)]
*M*_r_	334.80
Crystal system, space group	Orthorhombic, *P*2_1_2_1_2_1_
Temperature (K)	173
*a*, *b*, *c* (Å)	7.0614 (4), 11.0738 (6), 17.6541 (10)
*V* (Å^3^)	1380.49 (13)
*Z*	4
Radiation type	Mo *K*α
μ (mm^−1^)	1.61
Crystal size (mm)	0.58 × 0.25 × 0.11

Data collection	
Diffractometer	Bruker APEXIII CCD
Absorption correction	Multi-scan (*SADABS*; Bruker, 2017 ▸)
*T*_min_, *T*_max_	0.65, 0.70
No. of measured, independent and observed [*I* > 2σ(*I*)] reflections	21250, 3706, 2981
*R*_int_	0.078
(sin θ/λ)_max_ (Å^−1^)	0.728

Refinement	
*R*[*F* ^2^ > 2σ(*F* ^2^)], *wR*(*F* ^2^), *S*	0.028, 0.097, 1.33
No. of reflections	3706
No. of parameters	195
H-atom treatment	H atoms treated by a mixture of independent and constrained refinement
Δρ_max_, Δρ_min_ (e Å^−3^)	1.40, −2.47
Absolute structure	Flack *x* determined using 1080 quotients [(*I* ^+^)−(*I* ^−^)]/[(*I* ^+^)+(*I* ^−^)] (Parsons *et al.*, 2013 ▸)
Absolute structure parameter	0.013 (6)

Computer programs: *APEX3* and *SAINT* (Bruker, 2017 ▸), *SHELXT2014/5* (Sheldrick, 2015*a* ▸), *SHELXL2016/6* (Sheldrick, 2015*b* ▸), *Mercury* (Macrae *et al.*, 2020 ▸) and *SHELXTL* (Sheldrick, 2008 ▸).

## supplementary crystallographic information

### Crystal data

**Table:**

[Cu(C_11_H_11_NO_4_)(CH_4_O)(H_2_O)]	*D*_x_ = 1.611 Mg m^−^^3^
*M**_r_* = 334.80	Mo *K*α radiation, λ = 0.71073 Å
Orthorhombic, *P*2_1_2_1_2_1_	Cell parameters from 3748 reflections
*a* = 7.0614 (4) Å	θ = 3.5–27.2°
*b* = 11.0738 (6) Å	µ = 1.61 mm^−^^1^
*c* = 17.6541 (10) Å	*T* = 173 K
*V* = 1380.49 (13) Å^3^	Prism, green
*Z* = 4	0.58 × 0.25 × 0.11 mm
*F*(000) = 692	

### Data collection

**Table:**

Bruker APEXIII CCD diffractometer	3706 independent reflections
Radiation source: fine-focus sealed tube	2981 reflections with *I* > 2σ(*I*)
Detector resolution: 7.3910 pixels mm^-1^	*R*_int_ = 0.078
φ and ω scans	θ_max_ = 31.2°, θ_min_ = 2.3°
Absorption correction: multi-scan (*SADABS*; Bruker, 2017)	*h* = −10→10
*T*_min_ = 0.65, *T*_max_ = 0.70	*k* = −15→14
21250 measured reflections	*l* = −24→24

### Refinement

**Table:**

Refinement on *F*^2^	H atoms treated by a mixture of independent and constrained refinement
Least-squares matrix: full	*w* = 1/[σ^2^(*F*_o_^2^) + (0.0366*P*)^2^ + 0.3357*P*] where *P* = (*F*_o_^2^ + 2*F*_c_^2^)/3
*R*[*F*^2^ > 2σ(*F*^2^)] = 0.028	(Δ/σ)_max_ = 0.001
*wR*(*F*^2^) = 0.097	Δρ_max_ = 1.40 e Å^−^^3^
*S* = 1.33	Δρ_min_ = −2.47 e Å^−^^3^
3706 reflections	Extinction correction: SHELXL-2016/6 (Sheldrick, 2015*b*), Fc^*^=kFc[1+0.001xFc^2^λ^3^/sin(2θ)]^-1/4^
195 parameters	Extinction coefficient: 0.0061 (19)
0 restraints	Absolute structure: Flack *x* determined using 1080 quotients [(*I*^+^)-(*I*^-^)]/[(*I*^+^)+(*I*^-^)] (Parsons *et al.*, 2013)
Hydrogen site location: mixed	Absolute structure parameter: 0.013 (6)

### Special details

**Table:**

Geometry. All esds (except the esd in the dihedral angle between two l.s. planes) are estimated using the full covariance matrix. The cell esds are taken into account individually in the estimation of esds in distances, angles and torsion angles; correlations between esds in cell parameters are only used when they are defined by crystal symmetry. An approximate (isotropic) treatment of cell esds is used for estimating esds involving l.s. planes.

### Fractional atomic coordinates and isotropic or equivalent isotropic displacement parameters (Å^2^)

**Table:**

	*x*	*y*	*z*	*U*_iso_*/*U*_eq_	
Cu1	0.61538 (6)	0.62966 (4)	0.57142 (2)	0.01921 (15)	
N1	0.5954 (4)	0.5019 (3)	0.64462 (16)	0.0182 (6)	
O1	0.1357 (4)	0.6119 (3)	0.68853 (17)	0.0321 (7)	
C1	0.2945 (5)	0.6045 (4)	0.6605 (2)	0.0213 (8)	
O2	0.3562 (4)	0.6679 (3)	0.60469 (15)	0.0236 (6)	
C2	0.9302 (5)	0.4684 (4)	0.5454 (2)	0.0226 (8)	
O3	0.5999 (5)	0.7596 (3)	0.49822 (19)	0.0312 (7)	
H3	0.681 (8)	0.770 (5)	0.467 (3)	0.030 (14)*	
H2	0.510 (9)	0.813 (6)	0.497 (4)	0.043 (16)*	
C3	1.1020 (7)	0.4402 (4)	0.5083 (2)	0.0305 (9)	
H3A	1.143887	0.489273	0.468909	0.037*	
O4	0.8381 (4)	0.5673 (3)	0.52418 (16)	0.0238 (6)	
C4	1.2084 (6)	0.3414 (5)	0.5293 (3)	0.0357 (11)	
H4A	1.321203	0.325388	0.504001	0.043*	
C5	1.1509 (7)	0.2654 (4)	0.5873 (3)	0.0359 (11)	
H5A	1.225553	0.200181	0.601761	0.043*	
O5	0.5433 (4)	0.6871 (3)	0.76923 (17)	0.0270 (7)	
H5	0.630851	0.694020	0.739006	0.040*	
C6	0.9816 (7)	0.2880 (4)	0.6233 (3)	0.0295 (10)	
H6A	0.940205	0.235879	0.661122	0.035*	
O6	0.8220 (4)	0.7368 (3)	0.66221 (19)	0.0277 (7)	
H4	0.914663	0.694152	0.670341	0.043 (16)*	
C7	0.8696 (6)	0.3893 (3)	0.6037 (2)	0.0225 (8)	
C8	0.7002 (5)	0.4070 (3)	0.6473 (2)	0.0203 (8)	
H7A	0.663550	0.345351	0.679967	0.024*	
C9	0.4331 (5)	0.5140 (4)	0.6951 (2)	0.0205 (8)	
H8A	0.370634	0.435523	0.700865	0.025*	
C10	0.4885 (6)	0.5637 (4)	0.7740 (2)	0.0224 (8)	
H10A	0.373683	0.561185	0.805101	0.027*	
C11	0.6344 (7)	0.4869 (4)	0.8141 (2)	0.0310 (9)	
H11A	0.597899	0.403518	0.811274	0.046*	
H11B	0.642361	0.510986	0.866273	0.046*	
H11C	0.755515	0.497466	0.790346	0.046*	
C12	0.8794 (7)	0.8592 (4)	0.6574 (3)	0.0387 (10)	
H12A	0.990908	0.865077	0.626517	0.058*	
H12B	0.906569	0.889230	0.707250	0.058*	
H12C	0.779612	0.906256	0.635190	0.058*	

### Atomic displacement parameters (Å^2^)

**Table:**

	*U*^11^	*U*^22^	*U*^33^	*U*^12^	*U*^13^	*U*^23^
Cu1	0.0168 (2)	0.0205 (2)	0.0203 (2)	0.00153 (18)	0.00096 (18)	0.00462 (17)
N1	0.0179 (14)	0.0197 (16)	0.0170 (14)	0.0001 (13)	−0.0011 (13)	0.0003 (11)
O1	0.0178 (12)	0.0454 (18)	0.0330 (15)	0.0048 (13)	0.0040 (12)	0.0103 (13)
C1	0.0173 (16)	0.025 (2)	0.0212 (19)	−0.0012 (14)	−0.0032 (15)	0.0023 (15)
O2	0.0177 (12)	0.0286 (15)	0.0246 (14)	0.0036 (11)	0.0010 (12)	0.0074 (11)
C2	0.0219 (18)	0.025 (2)	0.0209 (18)	0.0039 (15)	−0.0024 (15)	−0.0038 (15)
O3	0.0224 (15)	0.0358 (17)	0.0354 (16)	0.0069 (14)	0.0069 (15)	0.0201 (13)
C3	0.027 (2)	0.037 (2)	0.028 (2)	0.003 (2)	0.006 (2)	−0.0015 (16)
O4	0.0224 (13)	0.0229 (14)	0.0261 (15)	0.0020 (11)	0.0049 (12)	0.0036 (11)
C4	0.025 (2)	0.046 (3)	0.036 (3)	0.0113 (19)	0.0019 (18)	−0.011 (2)
C5	0.036 (2)	0.037 (3)	0.034 (2)	0.018 (2)	−0.006 (2)	−0.0042 (19)
O5	0.0306 (16)	0.0244 (16)	0.0260 (16)	0.0016 (12)	0.0025 (13)	−0.0043 (12)
C6	0.036 (2)	0.025 (2)	0.028 (2)	0.0063 (18)	−0.0023 (19)	0.0003 (17)
O6	0.0183 (13)	0.0266 (16)	0.0384 (17)	0.0004 (11)	−0.0019 (13)	−0.0006 (13)
C7	0.0235 (17)	0.022 (2)	0.0225 (17)	0.0017 (16)	−0.0028 (16)	−0.0054 (14)
C8	0.0249 (18)	0.0161 (19)	0.0198 (18)	−0.0005 (14)	−0.0011 (15)	0.0001 (14)
C9	0.0175 (16)	0.022 (2)	0.0219 (18)	−0.0005 (13)	0.0023 (15)	0.0028 (14)
C10	0.0246 (19)	0.023 (2)	0.0193 (19)	−0.0008 (15)	0.0016 (16)	0.0007 (15)
C11	0.039 (2)	0.030 (2)	0.0239 (19)	0.005 (2)	−0.0079 (19)	0.0002 (16)
C12	0.042 (2)	0.031 (2)	0.043 (2)	−0.005 (2)	0.004 (2)	−0.0045 (19)

### Geometric parameters (Å, º)

**Table:**

Cu1—O6	2.471 (3)	C5—H5A	0.9300
Cu1—O4	1.910 (3)	O5—C10	1.423 (5)
Cu1—N1	1.922 (3)	O5—H5	0.8200
Cu1—O3	1.937 (3)	C6—C7	1.415 (6)
Cu1—O2	1.968 (3)	C6—H6A	0.9300
N1—C8	1.286 (5)	O6—C12	1.417 (5)
N1—C9	1.458 (5)	O6—H4	0.8200
O1—C1	1.228 (5)	C7—C8	1.436 (6)
C1—O2	1.286 (5)	C8—H7A	0.9300
C1—C9	1.528 (5)	C9—C10	1.548 (6)
C2—O4	1.327 (5)	C9—H8A	0.9800
C2—C3	1.414 (6)	C10—C11	1.512 (6)
C2—C7	1.418 (6)	C10—H10A	0.9800
O3—H3	0.81 (6)	C11—H11A	0.9600
O3—H2	0.87 (7)	C11—H11B	0.9600
C3—C4	1.378 (7)	C11—H11C	0.9600
C3—H3A	0.9300	C12—H12A	0.9600
C4—C5	1.387 (7)	C12—H12B	0.9600
C4—H4A	0.9300	C12—H12C	0.9600
C5—C6	1.376 (7)		
			
O4—Cu1—N1	95.03 (13)	C7—C6—H6A	119.4
O4—Cu1—O3	91.37 (14)	C12—O6—H4	109.5
N1—Cu1—O3	172.54 (15)	C6—C7—C2	119.9 (4)
O4—Cu1—O2	166.91 (13)	C6—C7—C8	116.3 (4)
N1—Cu1—O2	83.64 (13)	C2—C7—C8	123.8 (4)
O3—Cu1—O2	89.26 (13)	N1—C8—C7	124.8 (4)
C8—N1—C9	120.3 (3)	N1—C8—H7A	117.6
C8—N1—Cu1	125.8 (3)	C7—C8—H7A	117.6
C9—N1—Cu1	113.6 (2)	N1—C9—C1	108.7 (3)
O1—C1—O2	125.6 (4)	N1—C9—C10	112.6 (3)
O1—C1—C9	117.9 (4)	C1—C9—C10	106.8 (3)
O2—C1—C9	116.6 (3)	N1—C9—H8A	109.6
C1—O2—Cu1	115.3 (2)	C1—C9—H8A	109.6
O4—C2—C3	118.1 (4)	C10—C9—H8A	109.6
O4—C2—C7	124.5 (4)	O5—C10—C11	112.5 (3)
C3—C2—C7	117.4 (4)	O5—C10—C9	110.9 (3)
Cu1—O3—H3	122 (4)	C11—C10—C9	113.2 (3)
Cu1—O3—H2	124 (4)	O5—C10—H10A	106.6
H3—O3—H2	114 (5)	C11—C10—H10A	106.6
C4—C3—C2	121.2 (4)	C9—C10—H10A	106.6
C4—C3—H3A	119.4	C10—C11—H11A	109.5
C2—C3—H3A	119.4	C10—C11—H11B	109.5
C2—O4—Cu1	125.3 (3)	H11A—C11—H11B	109.5
C3—C4—C5	121.4 (4)	C10—C11—H11C	109.5
C3—C4—H4A	119.3	H11A—C11—H11C	109.5
C5—C4—H4A	119.3	H11B—C11—H11C	109.5
C6—C5—C4	119.0 (4)	O6—C12—H12A	109.5
C6—C5—H5A	120.5	O6—C12—H12B	109.5
C4—C5—H5A	120.5	H12A—C12—H12B	109.5
C10—O5—H5	109.5	O6—C12—H12C	109.5
C5—C6—C7	121.1 (4)	H12A—C12—H12C	109.5
C5—C6—H6A	119.4	H12B—C12—H12C	109.5

### Hydrogen-bond geometry (Å, º)

**Table:**

*D*—H···*A*	*D*—H	H···*A*	*D*···*A*	*D*—H···*A*
O3—H2···O4^i^	0.87 (7)	1.84 (7)	2.692 (4)	169 (6)
O3—H3···O2^ii^	0.81 (6)	1.89 (6)	2.687 (4)	167 (6)
O5—H5···O6	0.82	1.97	2.783 (4)	171
O6—H4···O1^iii^	0.82	1.84	2.653 (4)	175

Symmetry codes: (i) *x*−1/2, −*y*+3/2, −*z*+1; (ii) *x*+1/2, −*y*+3/2, −*z*+1; (iii) *x*+1, *y*, *z*.
